# Navigating around the Current Options to Preserve and Regenerate Meniscus: A Long Journey Still to Be Pursued

**DOI:** 10.3390/ijms23116057

**Published:** 2022-05-27

**Authors:** Berardo Di Matteo, Giuseppe Anzillotti, Elizaveta Kon

**Affiliations:** 1 IRCCS Humanitas Research Hospital, Rozzano, 20089 Milan, Italy; berardo.dimatteo@gmail.com (B.D.M.); elizaveta.kon@humanitas.it (E.K.); 2 Department of Biomedical Sciences, Humanitas University, Pieve Emanuele, 20090 Milan, Italy

The beginning of meniscal surgery was attributed to Annadale at the end of the 19th century: interestingly, he performed the first documented surgical repair of the meniscus [1]. Nevertheless, during the following century, the most common approach for meniscal tears has been partial or total meniscectomy, with increasing popularity after the advent of arthroscopy [2]. Only a few decades ago, the mere suspicion of a meniscal tear was sufficient to justify meniscectomy [3]. The short-term functional benefit and the pain relief offered by this procedure should be weighted against the long-term complications, particularly the onset and progression of osteoarthritis (OA), a common disease with one of the highest economic and social burdens, with costs up to 2.5 percent of gross domestic product in most countries [4,5]. Many studies demonstrated the fundamental role of meniscus in whole joint homeostasis, since OA changes can be found, at 10 years of evaluation, in 40% of lateral compartments and 24% of medial compartments where previous meniscectomy was performed [6]. Rongen et al. [7] rose awareness on the importance of the menisci, demonstrating a three-fold increase in arthroplasty procedures in patients who underwent previous meniscectomy. Therefore, the research of the last decades mainly focused on valid strategies in the field of meniscus preservation, starting from repair techniques to regenerative approaches. To accomplish this urgent need, it is fundamental to distinguish different types of meniscal lesions based on etiology: traumatic tears and degenerative changes are located on opposite sides of the spectrum of meniscal pathology. Traumatic tears are those which could be candidates, whenever possible, of suturing, employing the most modern techniques and equipment. On the other side, degenerative lesions are part of a way more complex chapter: in fact, similarly to other articular tissues, menisci can be affected by aging or degenerative changes, occurring even in young subjects, leading to dysfunction and structural impairment [8,9].

Based on these premises, it appears fundamental to understand when and whether damaged meniscal tissue removal is necessary, and when it is not. A recent systematic review analyzed the outcomes of surgical treatment compared to that of conservative treatment for painful degenerative meniscus. The study outlined how, in patients without symptoms of catching or locking, a proper regimen of physical exercise achieved successful results, and surgery could therefore be considered only as a second-line approach in non-responsive patients [10]. These findings were in line with the 2016 “ESSKA Meniscus Consensus Statement”, which suggested conservative treatment as the first-line therapy in the case of degenerative meniscus. Let’s consider again the well-known dilemma ‘To cut…or Not?’ [11]: every surgical decision must be made considering the present status but also the future of our patients. Therefore, whenever we cut, we should be aware that preserving and repairing are necessary to remain a healthy joint.

Although these two sides of the river (i.e., traumatic vs. degenerative etiology) appear more and more distant, the orthopedic practitioner always has the imperative need to preserve the torn menisci, or to timely address meniscal loss before the onset of irreparable consequences. In navigating the mare magnum of the available options for preventing further degeneration and for stimulating regeneration, the injection of biological products seems to be one of the most attractive alternatives. Ultrasound-guided meniscal injection appears to be a safe and feasible option to directly drive products such as platelet-rich plasma (PRP) or mesenchymal stem cells (MSCs) inside the damaged meniscus. Both preclinical and clinical studies have been performed: the potential benefits of PRP were exploited by Guenoun et al. [12], who treated 10 patients affected by degenerative meniscal tears using intralesional PRP injection and demonstrating, at 6-months follow-up, satisfactory functional outcomes. Similar results were presented by Özyalvac et al. on 15 patients with a mean 32 months’ follow-up, and a more recent trial confirmed that autologous conditioned plasma is able to provide good and stable outcomes up to 18 months follow-up [13]. Nevertheless, more studies must be conducted, to standardize the volume injected and the different preparation techniques [14]. 

The potential of mesenchymal cells was used in the field of the degenerative meniscus as well, but the optimal cell source and delivery method still lack consensus [15]. Their immunomodulating, anti-inflammatory, and pro-angiogenic effects were exploited by Malanga et al., who enrolled twenty patients affected by mild-moderate OA and degenerative meniscal tear: intra-meniscal and intra-articular injections of micro-fragmented adipose tissue were performed with notable KOOS and NPS scores improvement at 3, 6, and 12 months [16].

Beyond the use of ortho-biologic products, meniscal replacement still has its role in clinical practice. In 1984, the first meniscal allograft transplantation was performed [17], paving the way for meniscal replacement strategies [18]. Although several studies have confirmed the efficacy of meniscal transplantation even in the long term, the limited availability of allografts pushed the research towards the exploitation of new biomaterials to vicar the function of the meniscus. The animal models evidenced the safety of biomaterials and bio-engineered scaffolds, and underlined their role in tissue healing and whole joint preservation [19]. Anyway, only a few scaffolds were translated into clinical application: the collagen meniscal scaffold and polyurethane meniscal scaffold. Even if their results were encouraging [20], there is an urgent need of new technologies to treat meniscectomized patients, whose number has been dramatically increasing over time: novel strategies such as cell augmentation, growth factors addition, and 3D bioprinting may help in developing products able to replace meniscus and delaying the onset of OA [21].

This explains the rationale behind the Special Issue entitled ‘Strategies for Meniscus Preservation and Regeneration: From the Lab to Clinical Practice’. The title was broad, and the merging between laboratory researchers and clinicians is often challenging. We made this choice consciously, and committed to one solid belief: the need to approach a clinical request from different sides and perspectives, starting from the microscope, and ending in the orthopedic ward. Among the papers included in the Issue, an in vitro study evaluated the effect of different concentrations of oxygen in the neonatal pig meniscus, demonstrating the fundamental role of hypoxia in the fibro-chondrogenic differentiation [22]. Another in vitro trial analyzed the selection of meniscus progenitors just through their adhesion to fibronectin, opening new ways in the field of meniscal tissue-engineering [23]. The role of biomarkers was also taken into account: a rat animal model suggested some biomarkers as red flags to be studied in cases of meniscal tear, in order to identify the risk of progression to OA early [24].

As mentioned before, the delivery of different bioactive substances inside the joint is currently one of the most studied approaches: we included one systematic review which summarized all the current evidence of intra-articular drug delivery systems [25], which could be exploited to enhance the regenerative potential of novel scaffolds. Lastly, a pre-clinical study investigated new feasible ways to produce scaffolds seeded with meniscal cells and MSCs, and analyzed their potential application in clinical practice [26].

All the research involved, indeed, helped us to better understand the possible ways to preserve the meniscus and even regenerate it. A lot of work has been done in the last 20 years in the field of meniscal sparing techniques, intra-meniscal injections, scaffolds, and the application of ortho-biologic products, but the journey is still long and full of barriers to climb, especially in terms of identifying “patient’s specific” prognostic factors, such as anatomical and biochemical features, necessary to personalize our therapeutic strategies.

Perhaps the large variability of topics included may intimidate the reader, but we believed that this is the strength of our Special Issue, which should be regarded as a compass to navigate the surgeon in a flow of alternatives, with a panoramic view of all the work done so far and a comprehensive starting point for developing future ideas.

## References

[1] Di Matteo B., Tarabella V., Filardo G., Viganò A., Tomba P., Marcacci M. (2013). Thomas Annandale: The first meniscus repair. Knee Surg. Sports Traumatol. Arthrosc..

[2] Di Matteo B., Moran C.J., Tarabella V., Viganò A., Tomba P., Marcacci M., Verdonk R. (2016). A history of meniscal surgery: From ancient times to the twenty-first century. Knee Surg. Sports Traumatol. Arthrosc..

[3] Smillie I.S. (1967). The current pattern of internal derangements of the knee joint relative to the menisci. Clin. Orthop. Relat. Res..

[4] Peshkova M., Lychagin A., Lipina M., Di Matteo B., Anzillotti G., Ronzoni F., Kosheleva N., Shpichka A., Royuk V., Fomin V. (2022). Gender-Related Aspects in Osteoarthritis Development and Progression: A Review. Int. J. Mol. Sci..

[5] Roos H., Laurén M., Adalberth T., Roos E.M., Jonsson K., Lohmander L.S. (1998). Knee osteoarthritis after meniscectomy: Prevalence of radiographic changes after twenty-one years, compared with matched controls. Arthritis Rheum. Off. J. Am. Coll. Rheumatol..

[6] Verdonk R., Madry H., Shabshin N., Dirisamer F., Peretti G.M., Pujol N., Spalding T., Vedonk P., Seil R., Condello V. (2016). The role of meniscal tissue in joint protection in early osteoarthritis. Knee Surg. Sports Traumatol. Arthrosc..

[7] Rongen J.J., Rovers M.M., van Tienen T.G., Buma P., Hannink G. (2017). Increased risk for knee replacement surgery after arthroscopic surgery for degenerative meniscal tears: A multi-center longitudinal observational study using data from the osteoarthritis initiative. Osteoarthr. Cartil..

[8] Merkel K.H. (1980). The surface of human menisci and its aging alterations during age. A combined scanning and transmission electron microscopic examination (SEM, TEM). Arch. Orthop. Trauma Surg..

[9] Tsujii A., Nakamura N., Horibe S. (2017). Age-related changes in the knee meniscus. Knee.

[10] Giuffrida A., Di Bari A., Falzone E., Iacono F., Kon E., Marcacci M., Gatti R., Di Matteo B. (2020). Conservative vs. surgical approach for degenerative meniscal injuries: A systematic review of clinical evidence. Eur. Rev. Med. Pharmacol. Sci..

[11] Reider B. (2015). To Cut … or Not?. Am. J. Sports Med..

[12] Guenoun D., Magalon J., de Torquemada I., Vandeville C., Sabatier F., Champsaur P., Jacquet C., Olivier M. (2020). Treatment of degenerative meniscal tear with intrameniscal injection of platelets rich plasma. Diagn. Interv. Imaging.

[13] Di Matteo B., Altomare D., Garibaldi R., la Porta A., Manca A., Kon E. (2021). Ultrasound-Guided Meniscal Injection of Autologous Growth Factors: A Brief Report. Cartilage.

[14] Mariani E., Canella V., Berlingeri A., Bielli A., Cattini L., Landini M.P., Kon E., Marcacci M., Di Matteo B., Filardo G. (2015). Leukocyte presence does not increase microbicidal activity of Platelet-rich Plasma in vitro. BMC Microbiol..

[15] Rinonapoli G., Gregori P., Di Matteo B., Impieri L., Ceccarini P., Manfreda F., Campofreda G., Caraffa A. (2021). Stem cells application in meniscal tears: A systematic review of pre-clinical and clinical evidence. Eur. Rev. Med. Pharm. Sci..

[16] Malanga G.A., Chirichella P.S., Hogaboom N.S., Capella T. (2021). Clinical evaluation of micro-fragmented adipose tissue as a treatment option for patients with meniscus tears with osteoarthritis: A prospective pilot study. Int. Orthop..

[17] Wirth C.J., Peters G., Milachowski K.A., Weismeier K.G., Kohn D. (2002). Long-term results of meniscal allograft transplantation. Am. J. Sports Med..

[18] Condello V., Zdanowicz U., Di Matteo B., Spalding T., Gelber P.E., Adravanti P., Heuberer P., Dimmen S., Sonnery-Cottet B., Hulet C. (2019). Allograft tendons are a safe and effective option for revision ACL reconstruction: A clinical review. Knee Surg. Sports Traumatol. Arthrosc..

[19] Di Matteo B., Perdisa F., Gostynska N., Kon E., Filardo G., Marcacci M. (2015). Meniscal Scaffolds—Preclinical Evidence to Support their Use: A Systematic Review. Open Orthop. J..

[20] Veronesi F., Di Matteo B., Vitale N.D., Filardo G., Visani A., Kon E., Fini M. (2021). Biosynthetic scaffolds for partial meniscal loss: A systematic review from animal models to clinical practice. Bioact. Mater..

[21] Perdisa F., Filardo G., Di Matteo B., Marcacci M., Kon E. (2014). Platelet rich plasma: A valid augmentation for cartilage scaffolds? A systematic review. Histol. Histopathol..

[22] Canciani B., Herrera Millar V.R., Pallaoro M., Aidos L., Cirillo F., Anastasia L., Maria Peretti G., Modina S.C., Mangiavini L., Di Giancamillo A. (2021). Testing Hypoxia in Pig Meniscal Culture: Biological Role of the Vascular-Related Factors in the Differentiation and Viability of Neonatal Meniscus. Int. J. Mol. Sci..

[23] Korpershoek J.V., Rikkers M., de Windt T.S., Tryfonidou M.A., Saris D.B.F., Vonk L.A. (2021). Selection of Highly Proliferative and Multipotent Meniscus Progenitors through Differential Adhesion to Fibronectin: A Novel Approach in Meniscus Tissue Engineering. Int. J. Mol. Sci..

[24] Huang N.-C., Yang T.-S., Busa P., Lin C.-L., Fang Y.-C., Chen I.-J., Wong C.-S. (2021). Detection and Evaluation of Serological Biomarkers to Predict Osteoarthritis in Anterior Cruciate Ligament Transection Combined Medial Meniscectomy Rat Model. Int. J. Mol. Sci..

[25] Gambaro F.M., Ummarino A., Andón F.T., Ronzoni F., di Matteo B., Kon E. (2021). Drug Delivery Systems for the Treatment of Knee Osteoarthritis: A Systematic Review of In Vivo Studies. Int. J. Mol. Sci..

[26] Korpershoek J.V., Ruijter M.D., Terhaard B.F., Hagmeijer M.H., Saris D.B., Castilho M., Malda J., Vonk L.A. (2021). Potential of Melt Electrowritten Scaffolds Seeded with Meniscus Cells and Mesenchymal Stromal Cells. Int. J. Mol. Sci..

